# Towards Plant Synthetic Genomics

**DOI:** 10.34133/bdr.0020

**Published:** 2023-10-16

**Authors:** Yuling Jiao, Ying Wang

**Affiliations:** ^1^ State Key Laboratory of Protein and Plant Gene Research, School of Life Sciences, Peking University, Beijing 100871, China.; ^2^ Peking-Tsinghua Center for Life Sciences, Center for Quantitative Biology, Academy for Advanced Interdisciplinary Studies, Peking University, Beijing 100871, China.; ^3^ Peking University Institute of Advanced Agricultural Sciences, Shandong Laboratory of Advanced Agricultural Sciences in Weifang, Weifang, Shandong 261325, China.; ^4^ College of Life Sciences, University of Chinese Academy of Sciences, Beijing 100049, China.

## Abstract

Rapid advances in DNA synthesis techniques have allowed the assembly and engineering of viral and microbial genomes. Multicellular eukaryotic organisms, with their larger genomes, abundant transposons, and prevalent epigenetic regulation, present a new frontier to synthetic genomics. Plant synthetic genomics have long been proposed, and exciting progress has been made using the top-down approach. In this perspective, we propose applying bottom-up genome synthesis in multicellular plants, starting from the model moss *Physcomitrium patens*, in which homologous recombination, DNA delivery, and regeneration are possible, although further optimizations are necessary. We then discuss technical barriers, including genome assembly and plant transformation, associated with synthetic genomics in seed plants.

## Introduction

**Fig. 1.**
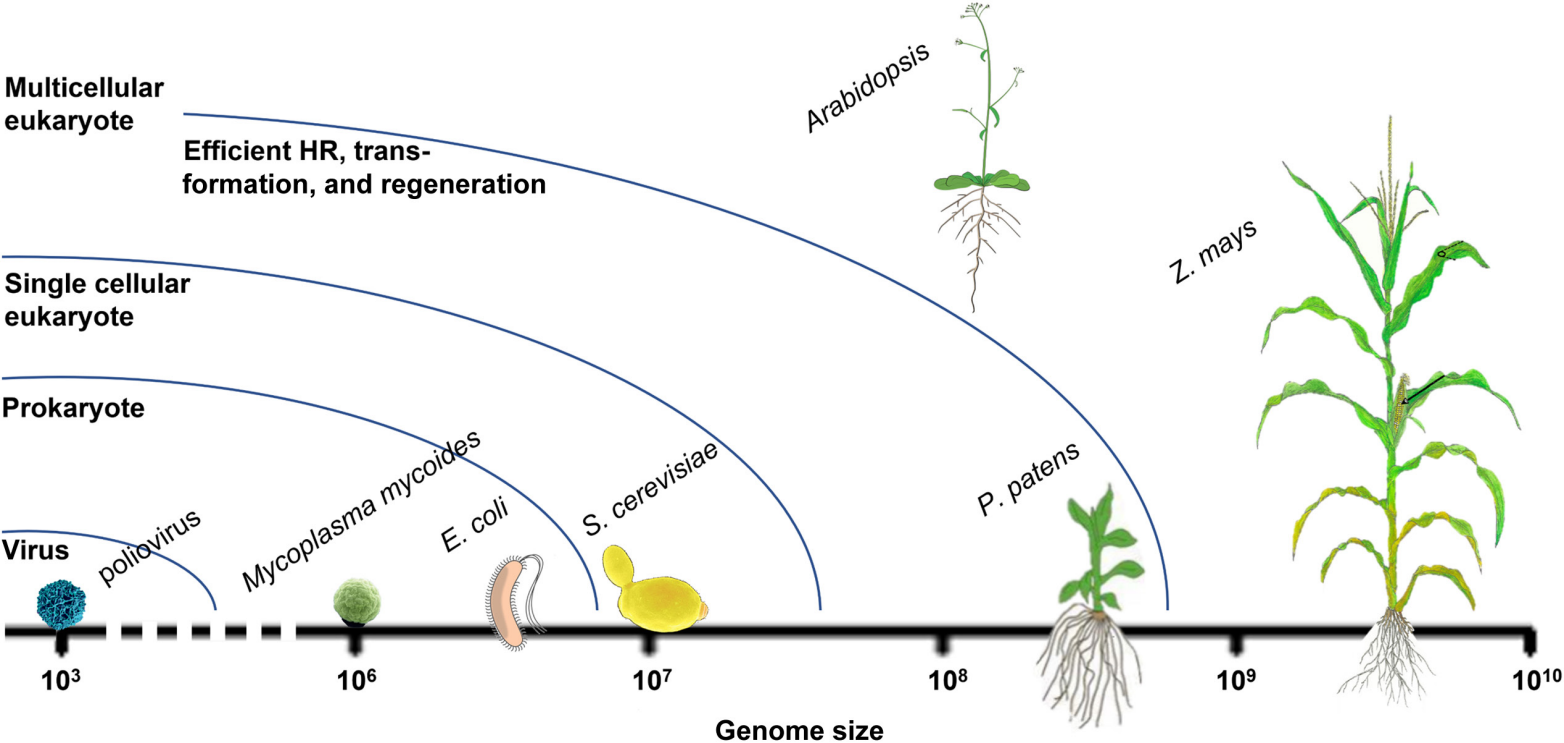
Comparison of genome size and other properties of existing and potential model species for synthetic genomics. HR, homologous recombination.

It has been well recognized that DNA carries the genetic information for living organisms. The ability to read DNA sequences has enormously expanded our understanding of biology. Following the sequencing of complete genomes, gene editing techniques have enabled us to modify the genome at desired sites, even in a high-throughput manner. More recently, advances in DNA synthesis techniques have made genome synthesis a new frontier [1]. Viral and bacterial genomes and yeast chromosomes have been engineered and reassembled using bottom-up approaches [2–7] (Fig. 1), enabling synthesis-based assays, such as accelerated evolution, multiplex gene deletions, and introduction or elimination of genetic codons. Despite the inevitable surge of interests in applying these technologies in more complex organisms, genome synthesis remains largely unexplored in multicellular organisms.

In viruses, bacteria, and yeast, the first step in genome synthesis is the design of synthetic genomes. There are multiple levels of consideration to be taken into account in genome design. A primary requirement is to distinguish between synthetic and native genomes. This is often achieved by altering or adding a few dozens of nucleotides, sometimes named watermarks, in intergenic regions that are tolerant to sequence changes [4]. PCRTags, as an alternative watermarking system, introduce synonymous changes into coding sequences within open reading frames, and enable polymerase chain reaction-based assay for not only the incorporation of synthetic sequences but also elimination of native sequences [2]. Additional designer alternations and inserted elements can provide new functions to synthetic genomes. In a synthetic yeast genome, a loxPsym site has been inserted after each stop codon, allowing whole-genome rearrangement upon Cre activation. With induced Cre expression, this technique, termed synthetic chromosome rearrangement and modification by loxPsym-mediated evolution, can restructure the synthetic genome, yielding highly variable structures and contents [2]. In bacteria and yeast, TAG stop codons have been replaced with TAA, reserving the TAG codon for a different function [2,6]. Repetitive sequences, such as transposable elements, have been eliminated in bacteria and yeast genome design, although they only represent a small portion of the genome [2,8].

Advances over the past two decades have enabled cost-effective DNA synthesis of ~150-nt oligonucleotides with high fidelity. Several in vitro and in vivo assembly methods have been developed to assemble building blocks at several to tens of kilobases. Among them, Gibson assembly method is widely used to in vitro assemble large building blocks [4]. Budding yeast has highly efficient homologous recombination and has been used as the chassis for large fragment assembly. The combined use of *Escherichia coli* and budding yeast has yielded hundred-kilobase level assembly efficiently [9]. Delivery of synthetic genomes is relatively simple in single cellular bacteria and budding yeast. Nevertheless, stepwise substitution, in which synthetic DNA chunks are iteratively introduced to replace the native counterpart by homologous recombination, has to be used during yeast genome synthesis [2].

Multicellularity is accompanied by the substantial increase in gene numbers and genome sizes. The variable and usually vast amounts of repetitive DNA derived from transposable elements account for an increase in genome sizes. Although transposable elements in certain chromosomal regions, such as centromeres and heterochromatin, have been found essential for chromosomal integrity and organismal survival [10], it is speculated that a substantial portion of transposable elements are dispensable. Natural selection is not effective enough to remove these transposable elements. Chromosomal-level elimination of transposable elements is expected to resolve whether a large amount of transposable elements can be simultaneously eliminated. In addition, epigenetic diversity expanded rapidly with multicellularity emergence, representing a new challenge to genome synthesis. It is unclear whether the epigenetic landscape in chromosomal-scale DNA chunks can be restored solely on the basis of sequence. Large DNA fragment transformation and assembly in multicellular organisms are difficult, and regeneration from transformed cells can be difficult, presenting additional hurdles.

## The Top-Down Approach for Plant Synthetic Genomics

Because of these difficulties, the abovementioned bottom-up approach has not been applied in multicellular organisms, including land plants. In plants, as well as most species studied so far, centromeres are difficult to synthesize because of their length (a few hundred kilobases or longer) and highly repetitive nature. Furthermore, sequences alone may be insufficient to de novo establish centromeres [11]. Therefore, a top-down approach has been proposed, in which a small chromosome, such as the supernumerary B chromosomes of maize, is simplified. By inserting telomeres, chromosome arms can be truncated to form a minichromosome [12]. By simultaneously inserting a loxP site, additional DNA fragments can be inserted through recombination, also termed gene stacking.

These pioneering studies have made exciting breakthroughs, and broad applications of the top-down strategy have to overcome certain limitations [13]. In particular, the constructed minichromosomes are not yet possible to isolate and transfer to another cell, or even another species, making sequence modifications tedious and time-consuming. In addition, chromosome truncation and assembly often rely on site-directed manipulation, which at least requires insertion of recombinase recognition sites to guide integration of large fragments delivered by *Agrobacterium*-mediated T-DNA vectors. Fortunately, prime editing and other new methods would facilitate site-specific modifications.

## Bottom-Up Synthetic Genomics in Moss

Advances in genome synthesis and chromosome assembly in yeast have shed new light in the field and have encouraged us to test an alternative bottom-up approach to building fully synthetic plant chromosomes in part or completely [13]. The early terrestrial plant *Physcometrium* (*Physcometrella*) *patens* is a well-established model organism for nonseed plants [14]. As a broadly used model in evolutionary developmental and cell biological studies, the *P. patens* genome has been fully sequenced [15]. Several characters have made *P. patens* a suitable testbed for bottom-up genome synthesis of multicellular organism. *P. patens* has efficient homologous recombination [16], providing a start point to explore large (>10-kb) fragment assembly. In addition, *P. patens* has high protoplast regeneration ability, making it possible to obtain viable plants from transformed cells. This property makes it possible to bypass *Agrobacterium*-mediated transformation. Protoplast can directly uptake large DNA fragments, which may integrate into desired genome loci through homologous recombination.

The *P. patens* genome is also an excellent system to test genome simplification as it has a relatively high transposable element (TE) content at ~60%, as well as sophisticated epigenetic landscape comparable to seed plants [15], so that the tested genome simplification strategies can be broadly translated and applied to seed plants. In addition, *P. patens* has been used for decades as a versatile chassis for synthetic biology to express recombinant therapeutic proteins and small natural products of high commercial value. All these characters make *P. patens* an ideal platform to explore bottom-up synthetic genomics in multicellular organisms.

Reconstructing the epigenetic landscape remains a challenge. For example, the epigenetic modification is likely key to centromere functions in *P. patens*. Sequence analysis indicates that *P. patens* centromeres are of comparable size to other plants and animals [15] but distinct from the point centromere of budding yeast, implying that epigenetic modification might be key to centromeric functions in *P. patens*. In cases where epigenetic modifications are not de novo established following DNA sequence guidance, epigenetic editing can be used to precisely modify the epigenetic profile [17].

## Challenges in Seed Plant Synthetic Genomics

Building synthetic chromosomes using the bottom-up approach in plant species other than *P. patens* needs to overcome additional barriers (Fig. 1). We list three outstanding challenges: (a) chromosome assembly, (b) functional centromere, and (c) transformation and regeneration.

Chromosome assembly has proven to be challenging. Whereas homologous recombination is widely utilized in bacteria and yeast genome assembly, its efficiency is extremely low in seed plants. In addition to inserting loxP or comparable recombination insertion sites [12], the CRISPR-CRISPR-associated protein (Cas) technology has been applied to create directional chromosome cleavage, which further induces segment translocation between chromosome arms [18]. The controlled mega–base pair range exchange between heterologous chromosome arms paves the way for future assembly in seed plants.

There are several criteria to establish functional centromeres. As mentioned above, centromeres in seed plants are not only large in size but also subject to complex epigenetic regulation that is key to their functions. Recent advances in maize and Arabidopsis have shown that a tethering approach that recruits centromeric histone H3 (CENH3) to a synthetic repeat array activates the formation of functional centromeres [19,20]. In maize, a LexA–CENH3 fusion protein organizes functional kinetochores at synthetic LexO repeat arrays, leading to chromosome breakages. Chromosome fragments form neochromosomes that can be stably inherited and further self-sustained in the absence of the LexA–CENH3 activator [19].

Although yeast and bacteria assembly of a plant chromosome is potentially possible, the chromosome will have to be transformed into a plant cell. *Agrobacterium*-mediated transformation, biolistic transformation, and protoplast transformation have their advantages and limitations. If protoplast transformation is used for its possibly higher tolerance to large DNA fragments, regeneration efficiency must be improved to obtain viable plants.

## Conclusions

Compared with animals, plants have fewer ethic issues and are more likely to regenerate from a single transformed cell. Hence, it is likely that plants will pioneer genome synthesis in multicellular organisms for testing aggressive deletion of repetitive sequences, epigenetic reconstruction in synthetic sequences, and optimizing design principles. It is easier to test-run genome design, synthesis, and assembly in *P. patens*, and experience accumulated will be very useful to be applied to seed plants, including crops. Knowledge obtained from this effort, in combination with top-down and bottom-up approaches in other species, will likely to teach us much about what is needed for a functional plant chromosome. Looking forward, these endeavors may also lead to other biotechnology breakthroughs other than providing vectors for large DNA chunks.

## References

[1] Zhang W, Mitchell LA, Bader JS, Boeke JD. Synthetic genomes. Annu Rev Biochem. 2020;89:77–101. 32569517 10.1146/annurev-biochem-013118-110704

[2] Richardson SM, Mitchell LA, Stracquadanio G, Yang K, Dymond JS, DiCarlo JE, Lee D, Huang CLV, Chandrasegaran S, Cai Y, et al. Design of a synthetic yeast genome. Science. 2017;355(6329):1040–1044. 28280199 10.1126/science.aaf4557

[3] Dymond JS, Richardson SM, Coombes CE, Babatz T, Muller H, Annaluru N, Blake WJ, Schwerzmann JW, Dai J, Lindstrom DL, et al. Synthetic chromosome arms function in yeast and generate phenotypic diversity by design. Nature. 2011;477(7365):471–476. 21918511 10.1038/nature10403 PMC3774833

[4] Gibson DG, Glass JI, Lartigue C, Noskov VN, Chuang RY, Algire MA, Benders GA, Montague MG, Ma L, Moodie MM, et al. Creation of a bacterial cell controlled by a chemically synthesized genome. Science. 2010;329(5987):52–56. 20488990 10.1126/science.1190719

[5] Zurcher JF, Robertson WE, Kappes T, Petris G, Elliott TS, Salmond GPC. Refactored genetic codes enable bidirectional genetic isolation. Science. 2022;378(6619):516–523. 36264827 10.1126/science.add8943 PMC7614150

[6] Isaacs FJ, Carr PA, Wang HH, Lajoie MJ, Sterling B, Kraal L, Tolonen AC, Gianoulis TA, Goodman DB, Reppas NB, et al. Precise manipulation of chromosomes in vivo enables genome-wide codon replacement. Science. 2011;333(6040):348–353. 21764749 10.1126/science.1205822 PMC5472332

[7] Fredens J, Wang K, de la Torre D, Funke LFH, Robertson WE, Christova Y, Chia T, Schmied WH, Dunkelmann DL, Beránek V, et al. Total synthesis of Escherichia coli with a recoded genome. Nature. 2019;569(7757):514–518. 31092918 10.1038/s41586-019-1192-5 PMC7039709

[8] Pósfai G, Plunkett G, Fehér T, Frisch D, Keil GM, Umenhoffer K. Emergent properties of reduced-genome *Escherichia coli*. Science. 2006;312(5776):1044–1046. 16645050 10.1126/science.1126439

[9] Jiang S, Tang Y, Xiang L, Zhu X, Cai Z, Li L, Chen Y, Chen P, Feng Y, Lin X, et al. Efficient *de novo* assembly and modification of large DNA fragments. Sci China Life Sci. 2022;65(7):1445–1455. 34939159 10.1007/s11427-021-2029-0

[10] Gemmell NJ. Repetitive DNA: Genomic dark matter matters. Nat Rev Genet. 2021;22(6):342. 10.1038/s41576-021-00354-8 33782602

[11] Birchler JA. Promises and pitfalls of synthetic chromosomes in plants. Trends Biotechnol. 2015;33(3):189–194. 25624060 10.1016/j.tibtech.2014.12.010

[12] Yu W, Lamb JC, Han F, Birchler JA. Telomere-mediated chromosomal truncation in maize. Proc Natl Acad Sci U S A. 2006;103(46):17331–17336. 17085598 10.1073/pnas.0605750103 PMC1859930

[13] Dawe RK. Charting the path to fully synthetic plant chromosomes. Exp Cell Res. 2020;390(1): 111951. 32151492 10.1016/j.yexcr.2020.111951

[14] Rensing SA, Goffinet B, Meyberg R, Wu SZ, Bezanilla M. The moss *Physcomitrium* (*Physcomitrella*) *patens*: A model organism for non-seed plants. Plant Cell. 2020;32(5):1361–1376. 32152187 10.1105/tpc.19.00828 PMC7203925

[15] Lang D, Ullrich KK, Murat F, Fuchs J, Jenkins J, Haas FB, Piednoel M, Gundlach H, van Bel M, Meyberg R, et al. The *Physcomitrella patens* chromosome-scale assembly reveals moss genome structure and evolution. Plant J. 2018;93(3):515–533. 29237241 10.1111/tpj.13801

[16] Schaefer DG, Zrÿd JP. Efficient gene targeting in the moss *Physcomitrella patens*. Plant J. 1997;11(6):1195–1206. 9225463 10.1046/j.1365-313x.1997.11061195.x

[17] Gjaltema RAF, Rots MG. Advances of epigenetic editing. Curr Opin Chem Biol. 2020;57:75–81. 32619853 10.1016/j.cbpa.2020.04.020

[18] Beying N, Schmidt C, Pacher M, Houben A, Puchta H. CRISPR–Cas9-mediated induction of heritable chromosomal translocations in Arabidopsis. Nat Plants. 2020;6(6):638–645. 32451449 10.1038/s41477-020-0663-x

[19] Dawe KR, Gent JI, Zeng Y, Zhang H, Fu F-F, Swentowsky KW, Kim DW, Wang N, Liu J, Piri RD. Synthetic maize centromeres transmit chromosomes across generations. Nat Plants. 2023;9(3):433–441. 36928774 10.1038/s41477-023-01370-8

[20] Teo CH, Lermontova I, Houben A, Mette MF, Schubert I. *De novo* generation of plant centromeres at tandem repeats. Chromosoma. 2013;122(3):233–241. 23525657 10.1007/s00412-013-0406-0

